# Evaluation of ankle brachial index, serum miR-103 and LP-PLA2 in the prognosis of acute ischemic stroke

**DOI:** 10.12669/pjms.40.4.8716

**Published:** 2024

**Authors:** Qing Chang, Fan Zhang, Qian Xue, Aixia Song

**Affiliations:** 1 Qing Chang, Department of Neurology, First Affiliated Hospital of Hebei North University, Zhangjiakou 075000, Hebei Province, P.R. China; 2 Fan Zhang, Department of Neurology, First Affiliated Hospital of Hebei North University, Zhangjiakou 075000, Hebei Province, P.R. China; 3 Qian Xue, Department of Neurology, First Affiliated Hospital of Hebei North University, Zhangjiakou 075000, Hebei Province, P.R. China; 4 Aixia Song, Department of Neurology, First Affiliated Hospital of Hebei North University, Zhangjiakou 075000, Hebei Province, P.R. China

**Keywords:** Ankle brachial index, Acute ischemic stroke, Microribonucleic acid-103, Lipoprotein associated phospholipase A2

## Abstract

**Objective:**

To explore the prognostic value of ankle brachial index (ABI), serum microribonucleic acid-103 (miR-103), and lipoprotein associated phospholipase A2 (LP-PLA2) indicators in patients with acute ischemic stroke (AIS).

**Methods:**

A retrospective analysis was conducted using the medical records of 202 patients with AIS admitted to the First Affiliated Hospital of Hebei North University from June 2019 to December 2022. Patients were divided into two groups based on their prognosis: the Poor-group (n=72) and the Good-group (n=130). Levels of ABI, serum miR-103, and LP-PLA2 indicators were compared between the two groups. Multivariate logistic regression analysis was used to analyze the independent risk factors for the poor prognosis in patients with AIS, and the receiver operating characteristic (ROC) curve was used to evaluate the predictive ability of ABI, serum miR-103, and LP-PLA2 levels on the prognosis of AIS.

**Results:**

Seventy two patients had a poor prognosis (35.6%) and 130 had a good prognosis (64.4%). The Poor-group had a higher proportion of elderly patients, patients with a history of diabetes and hypertension, abnormal ABI, and elevations in serum miR-103 and LP-PLA2 compared to the Good-group (P<0.05). Multivariate logistic regression analysis showed that abnormal ABI, and high levels of serum miR-103 and LP-PLA2 were independent risk factors for the poor prognosis. ROC curve provided a combined AUC of 0.862, which was higher than that of the individual ABI, serum miR-103, and LP-PLA2 curves, with values of 0.625, 0.749, and 0.696, respectively (P<0.05).

**Conclusions:**

Abnormal ABI, and high serum miR-103 and LP-PLA2 levels are independent risk factors for poor prognosis in AIS patients. They can be used as important indicators for predicting the prognosis of AIS.

## INTRODUCTION

Ischemic stroke present acutely and is the leading cause of disability worldwide and the second leading cause of death.^1^ According to the Global Stroke Fact Sheet 2022 released by the World Stroke Organization, 25% of people are estimated to have a stroke in their lifetime.^1^ Stroke is associated with a profound impact on the psychological and physical health of patients and their families, and a significant socioeconomic burden.^1^,^2^ About 20% of all transient cerebral ischemic stroke incidents are caused by carotid atherosclerosis, an inflammatory disease of the arterial system that is characterized by tunica intima damage, inflammatory response and formation of atherosclerotic plaque.^3^,^4^ Rupture of arterial atherosclerotic plaque can cause cardiovascular and cerebrovascular diseases, which can lead to myocardial infarction, or sudden death.^5^,^6^ Therefore, identifying risk factors of ischemic stroke and assessing their predictive value is crucial.

Ankle brachial index (ABI), which refers to the ratio of ankle/dorsalis pedis artery systolic pressure to brachial artery systolic pressure, is a reliable, convenient and non-invasive method, often used for the diagnosis of peripheral artery disease.^7^ The inflammatory marker lipoprotein associated phospholipase A2 (LP-PLA2) is also associated with the development of atherosclerosis and cardiovascular events and could be relevant in the prediction of acute ischemic stroke (AIS).^8^ Studies show that another factor, microribonucleic acid-103 (miR-103), is involved in the atherosclerotic process and damages endothelial cells by targeting long chain non-coding RNA-WDR59 and Kruppel-like factor four, increasing chemokine and proinflammatory factors.^9^ However, the prognostic value of ABI, LP-PLA2, and serum miR-103, alone or in combination, in patients with AIS is still unclear. This study aimed to evaluate the predictive ability of ABI, serum miR-103 and LP-PLA2 on the prognosis of patients with AIS.

## METHODS

Clinical records of 202 patients (140 males and 62 females) with AIS, treated in the First Affiliated Hospital of Hebei North University from June 2019 to December 2022 were retrospectively reviewed. Three months after the discharge, the prognosis of each patient was evaluated using the Modified Rankin Scale (MRS).^10^ MRS scores are scored from 0-6, with a >2 indicating poor prognosis and a score ≤ 2 indicating good prognosis. A total of 72 patients had poor prognosis and were designated as the Poor-group, while 130 patients had good prognosis and were designated as the Good-group.

### Inclusion criteria:

- Acute Ischemic Stroke diagnosis using the diagnostic criteria of the Chinese Guidelines for the Diagnosis and Treatment of Acute Ischemic Stroke (2018).^11^
- All cases were confirmed through cranial CT or MRI scans.
- All cases presented with AIS for the first time.
- Age ≥ 18 years old.
- Complete medical record information.

### Exclusion criteria:

- Presence of immune system disease or malignant tumor.
- Recurrent stroke, transient ischemic attack, subarachnoid hemorrhage, or cerebral hemorrhage.
- Complicated with severe liver and kidney dysfunction.

### Ethical Approval

The study was approved by the Ethics Committee of the First Affiliated Hospital of Hebei North University (Approval No.: W2023017; Date: 23-April, 2023).

### ABI detection

Before detection, patients were asked to lie supine for 15 minutes in a 20^0^C room. ABI was detected using a HEM-7201 intelligent electronic sphygmomanometer (produced by Omron Co., Ltd.). Ankle artery pressure detection was carried out 2-3 cm above the ankle joint at the dorsalis pedis artery. The sensor was placed at the pulsation point of the posterior tibial artery. Brachial artery pressure was detected using an inflatable blood pressure cuff, and the end of the rubber tube of the cuff was placed on the pulsation point of the brachial artery. The lower edge of the cuff was placed 2-3 cm from the cubital fossa. Blood pressure was measured in both upper arms, then in both ankles, three times at two-minute intervals. The average value of the three tests was calculated and the ABI (ankle artery pressure/brachial artery pressure) was determined. The lowest value of the ABI on both the left and right sides was determined, with an ABI<0.9 considered abnormal.^12^

### Detection of serum miR-103 and LP-PLA2

Venous blood (3mL) was collected and centrifuged at rate of 1000r/minute for 15 minutes to remove the supernatant. The samples were stored at -20^0^C for future use. Serum miR-103 and LP-PLA2 were detected by a double antibody sandwich enzyme-linked immunosorbent assay (ELISA). The kit was purchased from Shanghai Enzyme-linked Biotechnology Co., Ltd.

### Statistical analysis

SPSS25.0 software (SPSS Inc., Chicago, IL, USA) was used to process the data, and Chi-square test was used to compare categorical variables. Continuous variables were tested for normality by using Shapiro-Wilk test. Non-normal data were presented as median (interquartile range [IQR]) and compared using Mann Whitney U-test. A multivariate logistic regression model was used, along with the receiver operating characteristic (ROC) curve and area under curve (AUC) to evaluate the predictive ability of ABI, and serum miR-103 and LP-PLA2 as indicators of AIS. Significance was set at *P*<0.05.

## RESULTS

A total of 202 patients with AIS met the inclusion criteria. There were 50 patients with history of diabetes and 53 patients with history of hypertension. Seventy-two patients were found to have poor prognosis (35.6%) and 130 were found to have good prognosis (64.4%). In the Poor-group, there was a higher proportion of elderly patients, patients with a history of diabetes and hypertension, abnormal ABI, and elevations in serum miR-103 and LP-PLA2 compared to the Good-group (P<0.05) (Table-I). Multivariate logistic regression analysis showed that abnormal ABI, high miR-103 index and LP-PLA2 index were independent risk factors for poor prognosis in patients with AIS (*P*<0.05) (Table-II).

**Table-I:** Comparison of basic information and related indicators between two groups.

Factor	Poor-group (n=72)	Good-group (n=130)	χ^2^/Z	P
Male	47 (65.3%)	93 (71.5%)	0.854	0.355
Age (years)	65(62, 69)	62(59, 68)	-2.293	0.022
History of diabetes (Yes)	26 (36.1%)	24 (18.5%)	7.750	0.005
History of hypertension (Yes)	25 (34.7%)	28 (21.5%)	4.161	0.041
History of smoking (Yes)	31 (43.1%)	46 (35.4%)	1.156	0.282
History of coronary heart disease (Yes)	19 (26.4%)	25 (19.2%)	1.394	0.238
History of drinking (Yes)	32 (44.4%)	50 (46.2%)	0.688	0.407
Abnormal ABI (Yes)	28 (38.8%)	27 (13.8%)	16.524	<0.001
miR-103	2.55(2.20, 3.27)	2.15(1.80, 2.50)	-5.864	<0.001
LP-PLA2 (ng/mL)	235.00(207.25, 261.75)	214.00(188.75, 241.00)	-4.617	<0.001

**Table-II:** Multivariate logistic regression analysis of AIS prognosis.

Variable	B	S.E.	Wald χ^2^	P	OR	95%CI
Abnormal ABI	1.247	0.432	8.321	0.004	0.287	0.123~0.671
miR-103	2.217	0.395	28.979	<0.001	8.393	3.868~18.209
LP-PLA2	0.024	0.007	12.432	<0.001	1.024	1.011~1.038

***Note:*** B indicates partial regression system; S.E. indicates standard error; Wald χ^2^=(B/S.E.)^2^; OR is odds ratio; 95%CI is the confidence interval of OR

**Fig.1:**
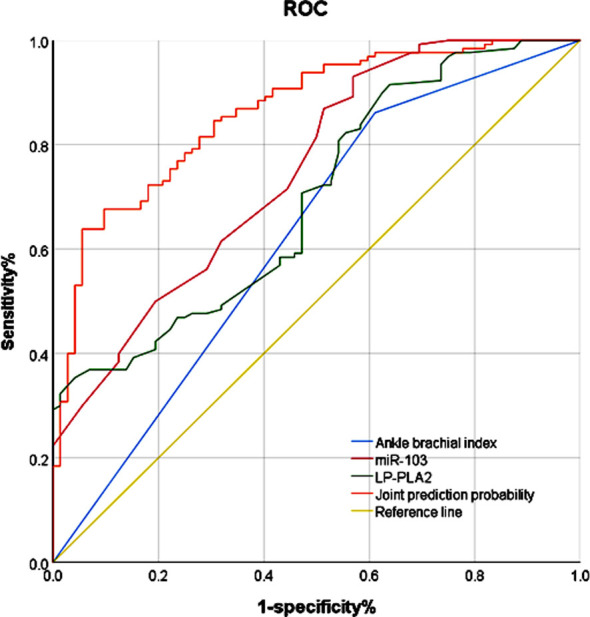
ROC curve of ABI, miR-103, LP-PLA2 in the prognosis of AIS.

ROC curve showed a higher AUC (0.862) when all three indicators were combined, compared to the AUC of individual ABI (0.625), serum miR-103 (0.749), and LP-PLA2 (0.696) (*P*<0.05). The specificity and sensitivity of all three indicators combined were 94.4% and 63.8%, respectively. (Table-III & Fig.1).

**Table-III:** Analysis of the predictive value of ABI, serum miR-103 and LP-PLA2 in the prognosis of AIS.

Indicators	AUC	Truncation value	95%Cl	Specificity (%)	Sensitivity (%)
ABI	0.625	0.500	0.542~0.709	38.9	86.2
miR-103	0.749	2.850	0.679~0.818	43.1	93.1
LP-PLA2	0.696	197.000	0.622~0.770	35.4	95.8
Joint testing	0.862	-	0.811~0.913	63.8	94.4

## DISCUSSION

This study showed that abnormal ABI, and high serum levels of miR-103 and LP-PLA2 are independent risk factors for the poor prognosis of AIS. ROC curves analysis demonstrated that the combination of these three indicators has a higher prediction ability than each indicator alone. We found that 35.6% (72/202) of patients with AIS in our study had a poor prognosis.

Since the etiology of AIS is complex, a simple and effective prediction method for identifying patients with the poor prognosis would be of great importance.^13^ Numerous studies have showed that ABI, a fast, inexpensive and non-invasive indicator of atherosclerosis, may be a predictor of stroke recurrence.^7^,^14^ Narrowing or occlusion of the peripheral arteries of the lower limbs as a result of aging, hypertension, diabetes, or other diseases, lead to a decrease in blood flow through the dorsalis pedis artery that is reflected by the changes in the ABI.^15^ The normal range of ABI is 0.9-1.3, whereas an ABI<0.9 suggests possible impairment of peripheral arterial flow in the lower limbs and cerebrovascular disease.^14^,^15^ The incidence of ABI<0.9 in patients with cerebral infarction is approximately 30% - 50%.^15^,^16^ A meta-analysis by Hong et al.^16^ that included 9404 patients found that low ABI increased the risk of composite outcomes (myocardial infarction, or stroke, or mortality), disability, and mortality. Similarly, Hajibandeh et al.^14^ examined 43 observational cohort studies with a total of 94,254 patients and found that ABI<0.9 was associated with an increase in cardiovascular and cerebrovascular incidence and mortality. In agreement with the outlined literature, ABI<0.9 in our study was identified as an independent risk factor for poor prognosis in patients with AIS.

MiRNAs are a class of non-coding, small molecule RNAs. One member of this class, miR-103, is known to increase the susceptibility of proliferating endothelial cells to mitotic aberrations induced by oxidized low-density lipoprotein through targeted expression of long chain, non-coding RNA WDR59, promoting vascular inflammation and atherosclerosis.^17^ Tang et al.^18^ analyzed 126 patients with AIS and found that upregulation of miR-103 expression was associated with the degree of neurological impairment and deterioration of neurological function. It seems that miR-103 may inhibit vascular endothelial growth factor and neovascularization and promote a decrease in neurological function.^19^ Our results are consistent with previous reports, and the excessive miR-103 in our study was identified an independent risk factor for poor prognosis in patients with AIS.

Increased LP-PLA2 was also found to be an independent risk factor for poor prognosis of AIS in our study, which is consistent with research by Wallentin et al.^20^ who found that an increase in LP-PLA2 positively correlated with cognitive impairment. LP-PLA2 is secreted by inflammatory cells, such as mature macrophages, and can promote the hydrolysis of oxidized phospholipids. It promotes inflammation and increases the occurrence of cardiovascular and cerebrovascular diseases by hydrolyzing oxidized phospholipids on the surface of low-density lipoprotein.^14^,^20^

In addition, a study by Berkovitch et al.^21^ that included 1047 patients hospitalized for acute coronary syndrome found that older age, diabetes and hypertension were risk factors for complications associated with AIS. In contrast, in our study older age and a history of diabetes and hypertension were not identified as independent risk factors of AIS prognosis. While the Poor-group did have a greater number of elderly patients with a history of diabetes and hypertension, the correlation was not statistically significant, possibly due to the smaller sample size and the selection bias.

Finally, the combination of ABI, miR-103, and LP-PLA2 resulted in a much higher AUC (0.862) compared to the individual AUC of these factors (0.625, 0.749, and 0.8696), which suggest that patients with AIS will present with a more severe response if they have a combination of abnormal ABI, and elevated serum miR-103 and LP-PLA2 indicators.^20^,^21^

### Limitation of the study

This is a retrospective study with small sample size conducted at a single center. Moreover, occurrence of adverse cardiovascular and cerebrovascular events was not assessed as there was no long-term follow-up analysis. Further multicenter, large-scale prospective studies are needed to improve the accuracy of the results.

## CONCLUSION

Abnormal ABI, and high serum miR-103 and LP-PLA2 are independent risk factors for poor prognosis of AIS. They can be used as important indicators for predicting the prognosis of patients with AIS.

### Authors’ contributions:

**QC:** Conceived and designed the study. **FZ**, **QX** and **AS:** Collected the data and performed the analysis. **QC:** Was involved in the writing of the manuscript and is responsible for the integrity of the study. All authors have read and approved the final manuscript.
